# Construction Notes

**Published:** 1894-09-01

**Authors:** 


### CONSTRUCTION NOTES.

**New Charnwood Forest Convalescent Home.**

This is in reality a country mansion available for the invalids among the working classes. It is large enough to receive forty to fifty patients, both male and female, and, with the exception of the dining-room, there is entirely separate acommodation for the two sexes. The whole building is supplied with almost every possible convenience for the health and comfort of the inmates, the bed-rooms being fitted with apparatus for warming when necessary, and specially constructed rooms are provided for patients who are suspected of having any contagious ailment. The cost of the whole work is estimated at about £6,000. The works were carried out from designs of Mr. G. H. Barrowcliffe, of Loughborough, Messrs. W. Moss and Sons being the chief contractors.

